# Multimodality lymphatic imaging of postoperative chylothorax in an infant with Noonan syndrome: a case report

**DOI:** 10.1186/s40001-020-00455-w

**Published:** 2020-11-04

**Authors:** Kay T. Pham, Duraisamy Balaguru, Varaha S. Tammisetti, Carlos J. Guevara, John C. Rasmussen, Rodrick C. Zvavanjanja, Robert Hanfland, Eva M. Sevick-Muraca, Melissa B. Aldrich

**Affiliations:** 1 Center for Molecular Imaging, The Brown Foundation Institute of Molecular Medicine, UT Health, 1825 Pressler St, Houston, TX 77030 USA; 2 Department of Pediatric Cardiology, McGovern Medical School At UTHealth, 6431 Fannin St, Houston, TX 77030 USA; 3 Department of Diagnostic & Interventional Imaging, 6431 Fannin St, Houston, TX 77030 USA; 4 Division of Pediatric Cardiothoracic Surgery, 6431 Fannin St, Houston, TX 77030 USA

**Keywords:** Chylothorax, Noonan syndrome, NIRFLI, MRL

## Abstract

**Background:**

Chylothorax is a rare complication of pediatric cardiac operations that occurs more frequently in children with Noonan syndrome, a genetic disorder associated with cardiac defects and lymphatic anomalies.

**Case presentation:**

We report a case of postoperative chylothorax in a 6-month-old infant with Noonan syndrome where multimodality lymphatic imaging guided management was followed. Drainage patterns of the lymphatic capillaries in the lower and upper extremities were visualized during near-infrared fluorescence lymphatic imaging (NIRFLI). Dynamic magnetic resonance lymphangiography (MRL) further identified the site of leakage in the thoracic duct and subsequently guided surgical intervention.

**Conclusions:**

Application of multimodality imaging allows for greater individualization of treatment and should be considered in patients with complex cases such as those with syndromes associated with a higher incidence of chylothorax. IRB Number: HSC-MS-13–0754, December 10, 2013

## Background

Noonan syndrome is a common (1 in 1000 to 2500) autosomal dominant genetic disorder with features including low-set, posteriorly rotated ears, hypertelorism, ptosis, short stature, cardiac abnormalities, webbed neck, and down-slanting palpebral fissures [1]. 15 responsible genes have been identified, with over half of Noonan cases associated with *PTPN11* variants [2]. Significant lymphatic abnormalities are associated with Noonan syndrome. Lymphedema may manifest as neck webbing and ptosis, or chylothorax, with extremity lymphedema, genital edema, and chylous reflux seen in 49–100, 82, and 73% of patients, respectively [2, 3]. Additionally, chylothorax can occur as a rare complication after heart surgery in children, thought to be secondary to surgical injury to mediastinal lymphatics. Chylothorax incidence is even higher with congenital lymphatic abnormalities, as seen in Noonan syndrome. Presently, postoperative chylothorax is managed using general anatomic knowledge, rather than personalized patient information. Decisions to implement thoracic duct ligation or pleurodesis are challenging, partly because of the difficulty in imaging central lymphatics and flow. Here, we describe the use of multimodality imaging, including near-infrared fluorescent lymphatic imaging (NIRFLI), to evaluate lymphatic flow, and dynamic magnetic resonance lymphangiography (MRL), to elucidate lymphatic anatomy, respectively, in a Noonan syndrome infant who developed chylothorax after heart surgery.

## Case presentation

A 6-month-old girl with confirmed PTPN11 mutation Noonan syndrome and cystic hygroma presented for tetralogy of Fallot repair. Informed parental consent was obtained for all procedures described hereafter.

Cardiac surgery consisted of a right ventricular (RV) outflow tract resection, pulmonary valvuloplasty, patch augmentation of the main pulmonary artery, and closure of atrial septal defect, leaving a small fenestration. Bilateral chylothorax developed postoperatively. Initially, 50–150 mL of fluid drained per day. Conservative therapy (medium-chain triglyceride/total parenteral nutrition) was instituted. This management strategy was ineffective so right and left pleurodeses via right thoracotomy were performed on postoperative days 24 and 25, respectively. During the procedure, there was no identification of the thoracic duct on the right side, suggesting anatomical variation of intrathoracic course. 50–150 mL/day continued to drain from the chest tube, so multimodality lymphatic imaging was performed to evaluate lymphatic flow contribution to chylothorax.

**Fig. 1:**
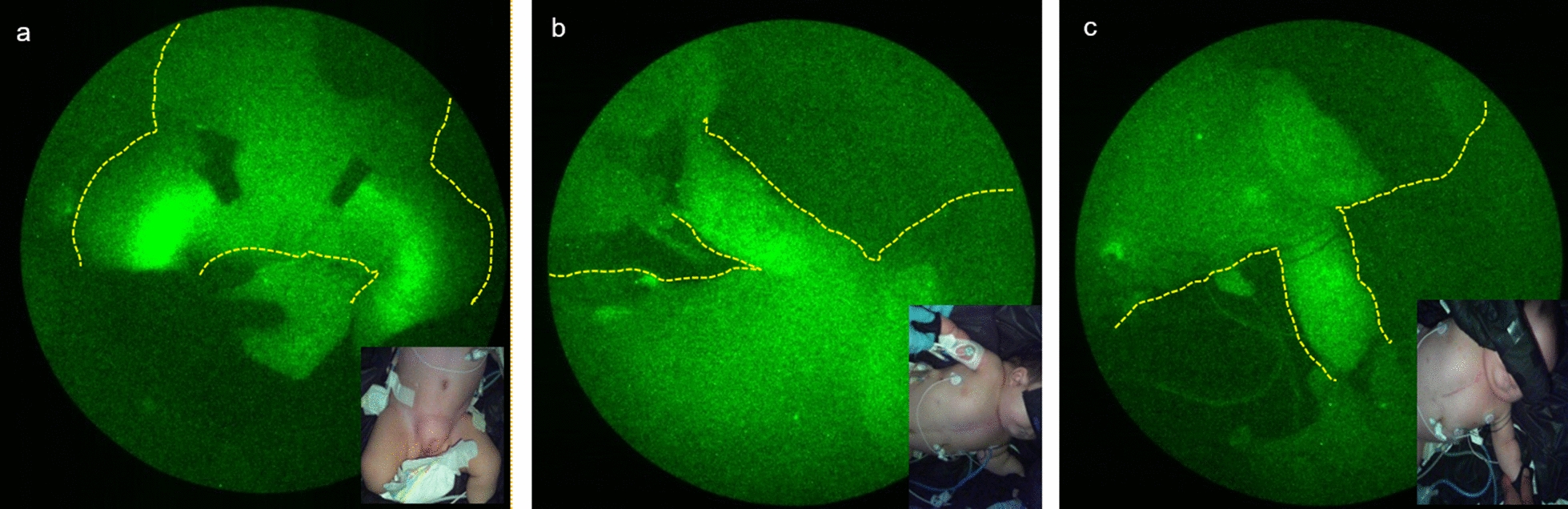
NIRFLI images. **a **Yellow arrows highlight pumping lymphatic vessels in both legs 60 min after intradermal injection of ICG in feet. Black tape covers brightly fluorescent inguinal lymph nodes. **b** Extravascular, non-pumping lymph (diffuse fluorescence) in right arm (**b**) and left arm (**c**) 20 min after injection in each hand. No fluorescence was noted in pleural spaces

**Fig. 2:**
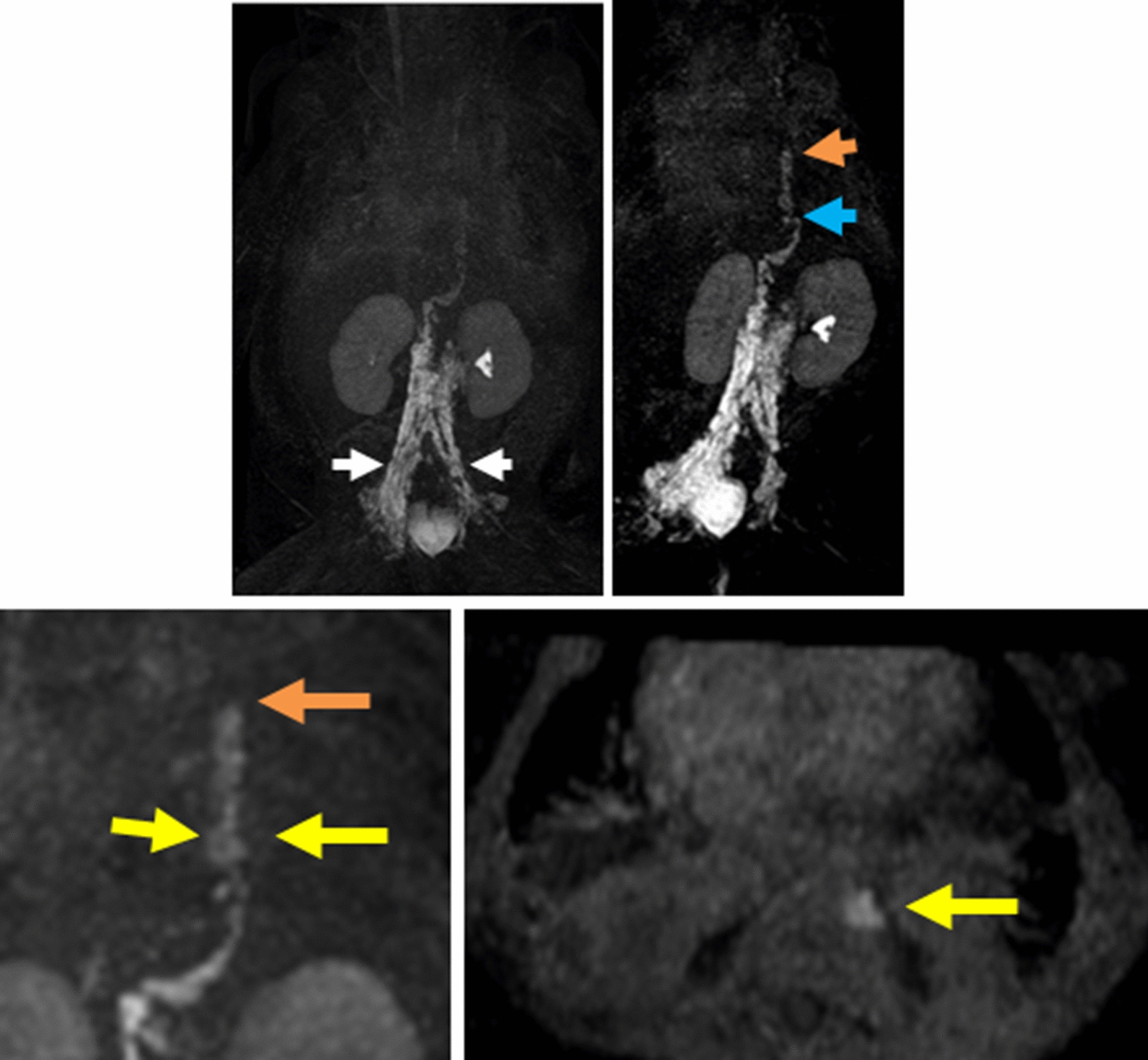
Selected serial MIP (maximal intensity projection) images of coronal 3D dynamic MR lymphangiogram performed at 1-min intervals for 30 min after inguinal lymph node injection of diluted gadolinium contrast. Progression of lymph flow into ectatic retroperitoneal lymphatics (white arrow), ectatic thoracic duct (blue arrow) with stricture of the thoracic duct (orange arrows) in mid-thoracic region and chyle leak into the pleural space at mid-thoracic level (yellow arrows)

For NIRFLI, described previously [4], performed under FDA (IND 122,035) and institutional IRB approval, intradermal injection of 0.05 cc of 0.25 mg/mL of indocyanine green (ICG; Akorn, Lake Forest, IL) to each dorsal foot was followed by observation for 1 h, and then injection to each hand was followed by observation for 20 min. Images revealed normal fluorescent dye uptake into leg lymphatics and inguinal lymph nodes; however, dye uptake into arm lymphatics was not observed—only diffuse, faint fluorescence (Fig. 1). This abnormal drainage pattern may have shown lymphedema consistent with lymphatic abnormalities commonly seen in Noonan syndrome patients, or that impaired drainage from the left-hand injection was due to high pressure in the central vein secondary to heart failure, considering that thrombotic occlusion of the subclavian veins had been ruled out by prior Doppler studies. Because NIRFLI was unable to identify a site of leakage, Coronal 3D T1 mDixon FFE dynamic MRL utilizing a 3 T Philips Ingenia MR scanner (Philips, Best, Netherlands) was then performed. 1.4 cc Gadavist (Bayer, Whippany, NJ), diluted 1:2 in normal saline, was injected into inguinal lymph nodes using ultrasound guidance. Firstly, MRL confirmed that the thoracic duct was located in the left mediastinum, explaining why the thoracic duct could not be identified during right thoracotomy. Opacification was promptly seen in the right retroperitoneal lymphatics, with chylolymphatic reflux in the left retroperitoneal lymphatics, and hiatus-level thoracic duct dilation. Furthermore, contrast leak from the thoracic duct proximal to the level of the T9 vertebra along the left paravertebral region, where thoracic duct caliber decreased, was observed (Fig. 2).

Based on the NIRFLI and MRL findings, conventional lymphangiography and transcatheter occlusion of the thoracic duct was attempted. Use of a water-based contrast, due to right-to-left shunting at the atrial septal level, was unsuccessful, due to inability to opacify the thoracic duct to cannulate. Thoracic duct ligation was performed via left thoracotomy guided by dynamic MRL. Chest tube drainage significantly dropped to 10–15 mL/day, but intermittent fevers, elevated white blood cell count (in the range of 50,000/cmm), low platelet count, blood culture growth of *Staphylococcus non-aureus*, and heparin-induced thrombocytopenia developed.

Four months after the initial cardiac surgery, a second heart surgery was performed to remove a right atrial thrombus and repair restenosis in the RV outflow tract and pulmonary arteries. Postoperatively, the patient developed severe ascites (drain output 600–1500 mL/day), and white blood cell count increased to 112,000/cmm. The patient was diagnosed with juvenile myelomonocytic leukemia, treated with allopurinol and 6-mercaptopurine. Severe ascites continued, and 32 days postoperatively, the patient succumbed to a bradycardic arrest.

## Discussion and conclusions

Imaging lymphatics in the setting of chylothorax is not common clinical practice. In this case, the conventional method of administering lipid-rich material to identify the site of a thoracic duct leak was contraindicated in our patient due to numerous cardiac complications. For this reason, we applied the latest lymphatic imaging technology to make informed surgical decisions.

NIRFLI provides an easy, safe bedside method without the use of anesthesia, to understand lymphatic flow patterns; however, a limitation is that central lymphatics are too deep to image. Therefore, we rely on appearance of fluorescence in the pleural spaces or the chest tube drainage fluid to determine chyle leakage. Although we were unable to use NIRFLI to determine a site of leakage in the thoracic duct, likely due to congenital lymphatic abnormalities or high CV pressure, the observations of impaired drainage could explain why conservative measures failed to resolve the chylothorax.

Dynamic MRL provides a reliable method to image central lymphatics with high resolution without the risk of systemic embolism of oil-based contrast material in patients with right-to-left shunts and can also provide a roadmap prior to conventional lymphangiography and intervention. In this case, however, conventional lymphangiography and intervention could not be utilized due to the inability to use oil-based contrast. Limitations of dynamic MRL include the need for anesthesia given the patient’s age and inguinal lymph node cannulation.

While thoracic duct ligation was considered to be successful in this patient, the second heart surgery, performed 4 months later, appears to have perturbed the fragile homeostasis achieved. Leukemia onset, frequently seen in Noonan patients [5], was an additional impediment in this case, and mortality was inevitable.

This case highlights the intricacies of treating Noonan infants with severe chylothorax. Other syndromes associated with chylothorax include Turner syndrome and Down syndrome [6]. Postoperative chylothorax is increasingly prevalent, perhaps due to increasing complexity of cardiac surgeries in children [7]. We believe that with continued application of lymphatic imaging modalities and acquisition of knowledge of various lymphatic abnormalities and patterns of injury, more individualized treatment of postoperative chylothorax in patients with these syndromes will be possible in the future.

## Data Availability

All data generated or analyzed during this study are included in this published article.
